# Detailed Molecular Interactions between Respiratory Syncytial Virus Fusion Protein and the TLR4/MD-2 Complex In Silico

**DOI:** 10.3390/v14112382

**Published:** 2022-10-28

**Authors:** Mao Akagawa, Tatsuya Shirai, Mitsuru Sada, Norika Nagasawa, Mayumi Kondo, Makoto Takeda, Koo Nagasawa, Ryusuke Kimura, Kaori Okayama, Yuriko Hayashi, Toshiyuki Sugai, Takeshi Tsugawa, Haruyuki Ishii, Hisashi Kawashima, Kazuhiko Katayama, Akihide Ryo, Hirokazu Kimura

**Affiliations:** 1 Department of Health Science, Graduate School of Health Sciences, Gunma Paz University, Takasaki-shi 370-0006, Japan; 2 Advanced Medical Science Research Center, Gunma Paz University Research Institute, Shibukawa-shi 377-0008, Japan; 3 Department of Clinical Engineering, Faculty of Medical Technology, Gunma Paz University, Takasaki-shi 370-0006, Japan; 4 Department of Virology III, National Institute of Infectious Diseases, Musashimurayama-shi, Tokyo 208-0011, Japan; 5 Department of Pediatrics, Graduate School of Medical Science, Chiba University, Chiba-shi 260-8670, Japan; 6 Department of Bacteriology, Graduate School of Medicine, Gunma University, Maebashi-shi 371-8514, Japan; 7 Department of Nursing Science, Graduate School of Health Science, Hiroshima University, Hiroshima-shi 734-8551, Japan; 8 Department of Pediatrics, School of Medicine, Sapporo Medical University, Sapporo-shi 060-8543, Japan; 9 Department of Respiratory Medicine, School of Medicine, Kyorin University, Mitaka-shi, Tokyo 181-8611, Japan; 10 Department of Pediatrics and Adolescent Medicine, Tokyo Medical University, Shinjuku-ku, Tokyo 160-0023, Japan; 11 Laboratory of Viral Infection Control, Graduate School of Infection Control Sciences, Ōmura Satoshi Memorial Institute, Kitasato University, Minato-ku, Tokyo 108-8641, Japan; 12 Department of Microbiology, School of Medicine, Yokohama City University, Yokohama-shi 236-0004, Japan

**Keywords:** respiratory syncytial virus, Toll-like receptor 4, myeloid differentiation factor-2, in silico, docking simulation

## Abstract

Molecular interactions between respiratory syncytial virus (RSV) fusion protein (F protein) and the cellular receptor Toll-like receptor 4 (TLR4) and myeloid differentiation factor-2 (MD-2) protein complex are unknown. Thus, to reveal the detailed molecular interactions between them, in silico analyses were performed using various bioinformatics techniques. The present simulation data showed that the neutralizing antibody (NT-Ab) binding sites in both prefusion and postfusion proteins at sites II and IV were involved in the interactions between them and the TLR4 molecule. Moreover, the binding affinity between postfusion proteins and the TLR4/MD-2 complex was higher than that between prefusion proteins and the TLR4/MD-2 complex. This increased binding affinity due to conformational changes in the F protein may be able to form syncytium in RSV-infected cells. These results may contribute to better understand the infectivity and pathogenicity (syncytium formation) of RSV.

## 1. Introduction

Respiratory syncytial virus (RSV), which belongs to the genus Orthopneumovirus of Family Pneumoviridae, causes various respiratory illnesses, such as bronchitis, bronchiolitis, and pneumonia [1]. Moreover, RSV potentially causes fatal bronchiolitis/pneumonia in vulnerable populations, including infants, the elderly, and immunocompromised patients [2,3,4]. Thus, this viral infection is a major infectious disease burden, along with influenza [5,6,7].

RSV consists of two major antigens: fusion protein (F protein) and attachment glycoprotein (G protein) [1]. F protein plays essential roles in host cell infection and syncytium formation [8]. Thus, this antigen is associated with the pathogenesis of the virus [9]. Moreover, two types of F proteins are confirmed: prefusion and postfusion proteins [1]. The prefusion type changes into the postfusion type with drastic conformational changes [10,11]. The prefusion and postfusion proteins also show distinct antigenicity [12], but the molecular interactions among syncytium formation, prefusion proteins, and postfusion proteins are unknown.

The F protein has antigenic domains defined by monoclonal antibody (mAb) competition and structural research of F protein-mAb complexes [13]. Moreover, the neutralizing antibody (NT-Ab)-binding sites are divided into six non-overlapping regions (sites Ø, I, II, III, IV, and V) on the prefusion protein [14]. Sites II and IV are displayed on the postfusion protein surface, and specific mAbs against these sites have been used in clinical practice [15]. However, the precise mechanisms by which these antigenic sites cause RSV infection in host cells have not been elucidated.

More than 10 types of Toll-like receptors have been found to be closely associated with human innate immunity [16]. Among them, TLR4 (CD20) is mainly recognized in various substances, including bacterial lipopolysaccharides (LPSs) and virus glycoproteins, leading to innate immunity [17]. Moreover, TLR4 is a ligand for the RSV F protein [1,18]. However, the detailed molecular interactions between the RSV F protein and TLR4 remain unclear.

Another molecule, myeloid differentiation factor-2 (MD-2), forms a complex with TLR4 (TLR4/MD-2 complex) [19,20]. MD-2 molecules can bind to LPS and activate the signaling pathways, including myeloid differentiation factor 88 (MyD88), resulting in a response against bacterial infections [21,22]. However, the molecular interactions between this molecule and the F protein are unknown.

Recent progress in bioinformatics technologies, i.e., the docking simulation method, allows us to analyze detailed protein–protein interactions. Thus, to better understand the molecular interactions between the RSV F protein and TLR4, detailed docking simulation analyses were performed.

## 2. Materials and Methods

### 2.1. Protein Preparation

Three-dimensional (3D) structures of RSV prefusion protein (PDBID: 4JHW), postfusion protein (PDBID: 3RKI), and the human TLR4/MD-2/LPS complex (PDBID: 3FXI) were obtained from Protein Data Bank Japan (PDBJ) (https://pdbj.org/, accessed on 16 September 2021). Subsequently, the 3D structures of TLR4/MD-2 and LPS were separated from the TLR4/MD-2/LPS complexes. Based on a previous report, six antigenic sites in the prefusion protein were identified: site Ø: Ser62–Leu96 and Leu195–Asn227; site I: Asn27–Leu45, Pro312–Thr318, and Glu378–Pro389; site II: Asn254–Asn277; site III: Ser46–Thr54, Val301–Thr311, Asn345–Phe352, and Cys367–Glu378; site IV: Cys422–Gly471; and site V: Ser55–Leu61, Ser146–Asp194, and Ser287–Val300 [23].

### 2.2. Protein–Protein Docking and Optimal Docking Model Selection

We employed the HDOCK web server (http://hdock.phys.hust.edu.cn/, accessed on 1 October 2021) to perform molecular docking between the F protein and TLR4/MD-2. Six antigenic sites (Ø and I–V) in the prefusion protein were specified as the binding sites of the present docking simulation [24]. These binding site residues were not designated in TLR4/MD-2. For the prefusion protein, the top 20 docking models were determined from the generated models at each antigenic site based on the docking score of HDOCK, and a total of 120 docking models were created. In addition to HDOCK, two independent scoring functions, HawkDock (http://cadd.zju.edu.cn/hawkdock/, accessed on 15 February 2022) and PPI-Affinity (https://protdcal.zmb.uni-due.de/PPIAffinity/BA/1219/, accessed on 16 September 2022), were used to rescore the docking models generated by HDOCK [25,26]. The optimal models for the prefusion protein were selected based on the HDOCK, HawkDock, and PPI-Affinity ranking. Subsequently, the binding site in the postfusion protein docking simulation was specified from two antigenic sites (II and IV), which correspond to the specified binding site of the complex models selected in the prefusion docking simulation. Finally, docking models were created using HDOCK, and optimal models were determined in the postfusion protein, as described above. Next, the 3D protein–protein interactions were visualized using PyMOL 2.3.4. Detailed molecular interactions, intermolecular distances, and interacting residues were also analyzed using the PDBsum server (http://www.ebi.ac.uk/thornton-srv/databases/pdbsum/, accessed on 20 September 2022) [27].

### 2.3. Calculation of the Binding Affinity

The binding affinity was calculated using the HADDOCK web server (https://alcazar.science.uu.nl/services/HADDOCK2.2/, accessed on 20 September 2022), which uses amino acid residue contact-based statistical functions to predict the binding affinity. This shows that the contributions to binding affinity are inter-residue contacts (ICs) and the noninteracting surface (NIS) [28,29]. Based on these theories, the core formula for binding affinity calculation implemented by HADDOCK was as follows:

ΔGpredicted = −0.09459 ICscharged/charged − 0.10007 ICscharged/apolar + 0.19577 ICspolar/polar − 0.22671 ICspolar/apolar + 0.18681 %NISapolar + 0.13810 %NIScharged − 15.9433(1)

The %NIS represents the percentage of polar, apolar, and charged residues on NIS [28,29]. The residues were categorized according to a previous report as follows: polar: Cys, His, Asn, Gln, Ser, Thr, and Trp; apolar: Ala, Phe, Ile, Met, Pro, Val, Leu, and Tyr; and charged: Glu, Asp, Lys, and Arg [30].

### 2.4. Validation of the Present Docking Simulation

To validate the reliability of the present docking simulation, the docking model and its native geometry were compared. However, as no structure of a complex of the F protein from any virus and TLR4 has been registered in the Protein Data Bank, we could not perform redocking between the F protein and TLR4. Thus, we used the TLR4/MD-2/LPS complex and RSV prefusion protein/antibody complex for this validation approach.

First, a TLR4/MD-2/LPS complex model was constructed through docking simulations between TLR4/MD-2 and LPS using HDOCK. The HDOCK best-scored model was selected from the docking models. Subsequently, the molecular interactions and interacting sites between the docking model and the X-ray crystallography structure of the TLR4/MD-2/LPS complexes analyzed by PDBsum were compared.

Next, the 3D structure of the RSV prefusion protein/antibody CR9501/motavizumab complex model (PDBID: 6OE5) was downloaded from the PDBJ. Then, the 3D structures of the prefusion protein, antibody CR9501, and motavizumab were separated from the complex model to perform a redocking simulation between the prefusion protein and antibody CR9501. The molecular interactions and interacting sites between the docking models generated by HDOCK and the Cryo-electron microscopy (cryo-EM) structure of the prefusion protein/antibody CR9501 complexes were compared, as described above.

Furthermore, we evaluated the reliability of the interacting sites in the selected docking models using ScanNet (http://bioinfo3d.cs.tau.ac.il/ScanNet/, accessed on 11 October 2022), which predicts the protein-binding sites from a structure and shows its binding probability based on a deep learning approach [31]. The interacting sites between the present docking models and those predicted by ScanNet were also compared. We then evaluated the percentage correspondence between the interacting sites of the docking model and sites with a binding probability > 0.5 as calculated by ScanNet.

## 3. Results

### 3.1. Determination of Suitable Structures among the Candidates

**Figure 1:**
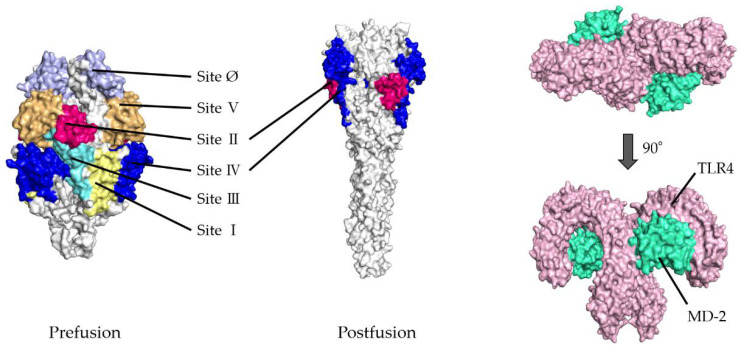
Illustration showing three-dimensional (3D) structures of respiratory syncytial virus (RSV) fusion proteins and the Toll-like receptor 4/myeloid differentiation factor-2 (TLR4/MD-2) complexes. Six antigenic sites in RSV prefusion protein are depicted as follows: site Ø (light purple), site I (light yellow), site II (red), site III (light blue), site IV (dark blue), and site V (light orange). RSV postfusion protein possesses sites II and IV. The TLR4 and MD-2 are shown in pink and light green, respectively.

- To validate the docking simulation, the docking models generated by HDOCK were rescored using HawkDock and PPI-Affinity. As shown in Table 1(a,b), the top five ranked docking models for the prefusion protein were all models in which sites II and IV were designated as the binding site. Thus, the best-scored models in sites II and IV were determined as the optimal models in the present docking simulation, respectively. The rank of the selected model in site II was first, twenty-seventh, and nineth, and in site IV, second, eighth, and tenth, based on the HDOCK, HawkDock, and PPI-Affinity scores, respectively, in 120 docking models. Similarly, in postfusion proteins, the optimal models were ranked and selected from among the top 20 docking models based on the HDOCK, HawkDock, and PPI-Affinity scores-site II: first, third, and third, respectively; site IV: first, third, and third, respectively. To understand the 3D structures of the F proteins (prefusion/postfusion) and TLR4/MD-2 complex easily, the natural structures are illustrated in Figure 1.

### 3.2. Molecular Interactions between Prefusion Proteins and TLR4/MD-2

**Figure 2:**
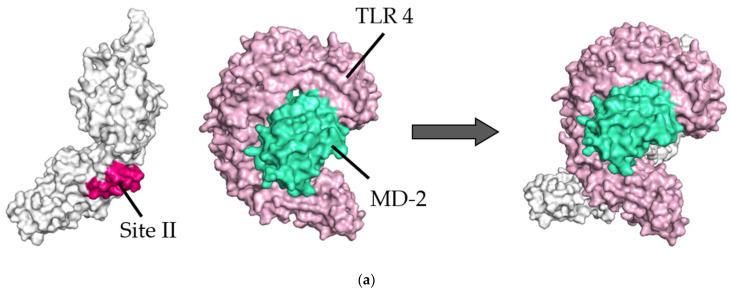

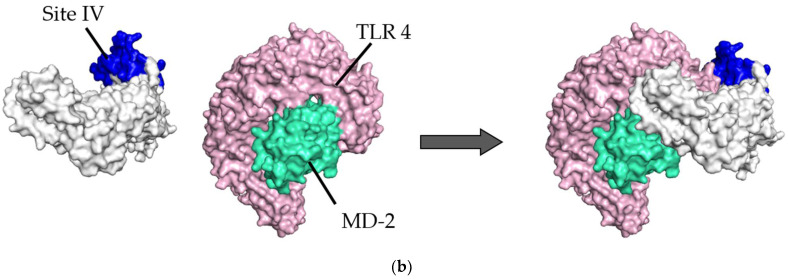
Diagram of binding conformations between respiratory syncytial virus (RSV) prefusion proteins and Toll-like receptor 4/myeloid differentiation factor-2 (TLR4/MD-2). (**a**) Three-dimensional (3D) structures of RSV prefusion proteins, TLR4/MD-2 complexes, and the protein–protein docking models in which site II was designated as the binding site. (**b**) 3D structures of RSV prefusion proteins, TLR4/MD-2 complexes, and the protein–protein docking models in which site IV was specified as the binding site. Sites II and IV in RSV fusion proteins are shown in red and dark blue, respectively. The TLR4 and MD-2 are colored pink and light green, respectively.

**Figure 3:**
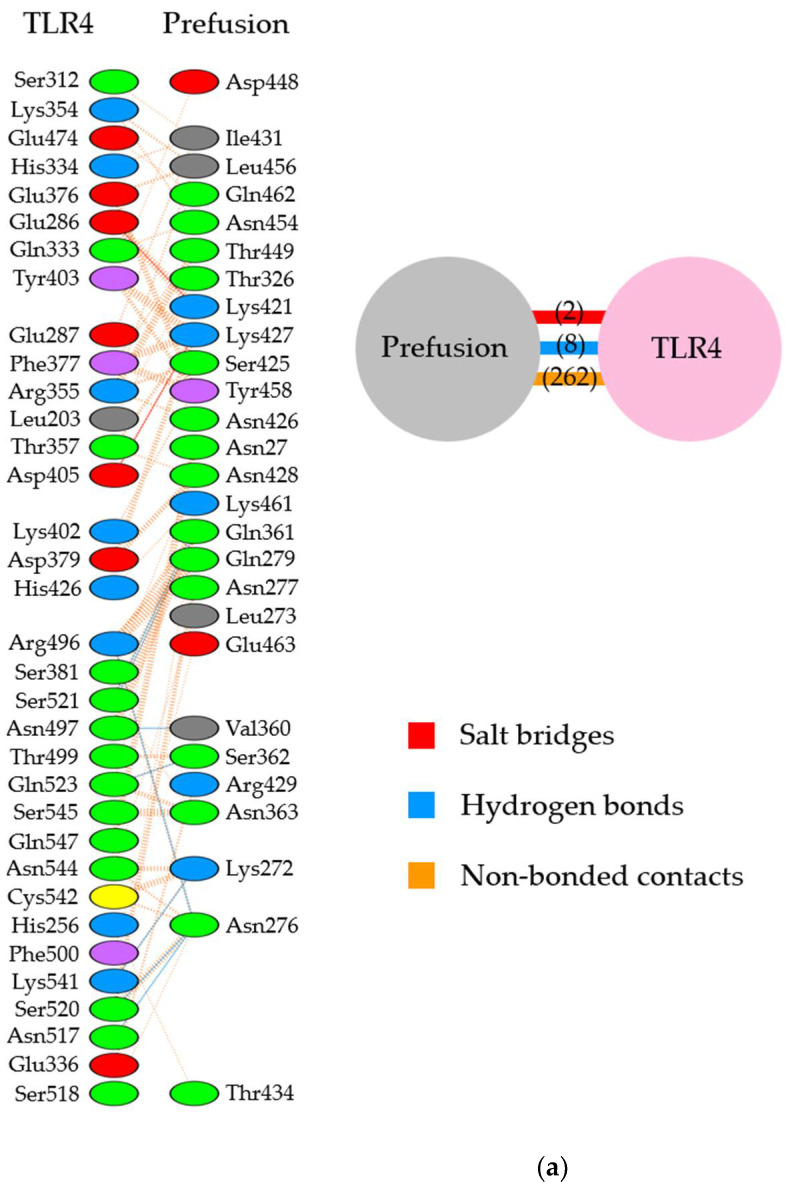

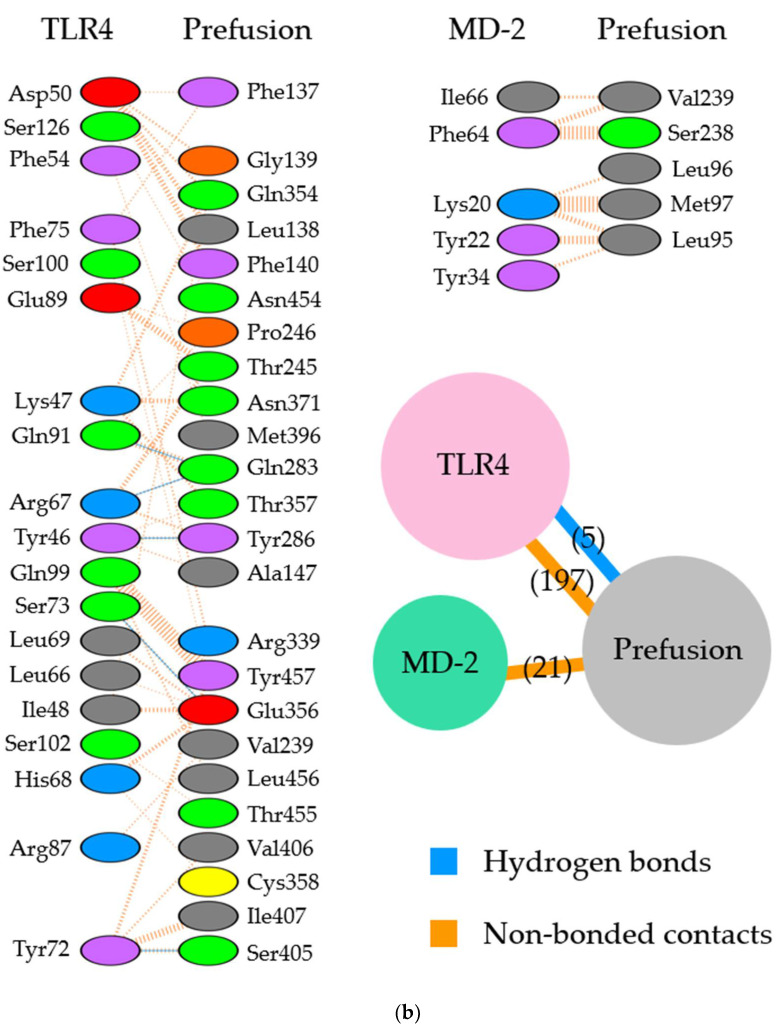
Illustration of the molecular interactions between respiratory syncytial virus (RSV) fusion proteins and Toll-like receptor 4/myeloid differentiation factor-2 (TLR4/MD-2). (**a**) The molecular interactions between RSV prefusion proteins in which site II was designated as the binding site and TLR4/MD-2 complexes. (**b**) The molecular interactions between RSV prefusion proteins in which site IV was specified as the binding site and TLR4/MD-2 complexes. The molecular interactions are depicted as follows: salt bridge (red), hydrogen bonds (blue), and non-bonded contacts (orange). The number on lines represents the number of bonds in the 2D protein diagram.

**Figure 4:**
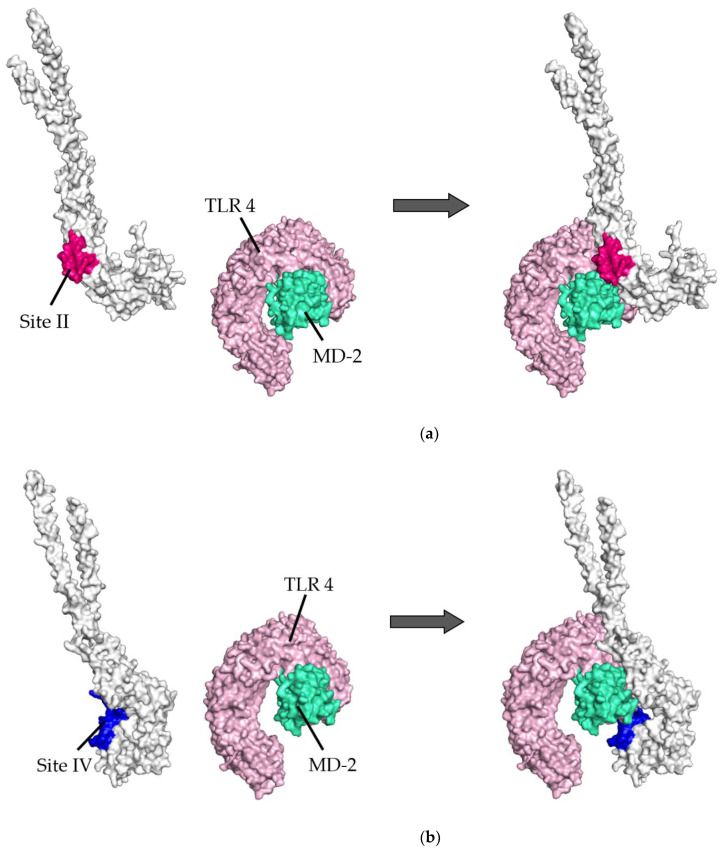
Diagram of binding conformations between respiratory syncytial virus (RSV) postfusion proteins and Toll-like receptor 4/myeloid differentiation factor-2 (TLR4/MD-2). (**a**) 3D structures of RSV postfusion proteins, TLR4/MD-2 complexes, and the protein–protein docking models in which site II was designated as the binding site. (**b**) 3D structures of RSV postfusion proteins, TLR4/MD-2 complexes, and the protein–protein docking models in which site IV was specified as the binding site. Sites II and IV in RSV fusion proteins are shown in red and dark blue, respectively. The TLR4 and MD-2 are colored pink and light green, respectively.

**Figure 5:**
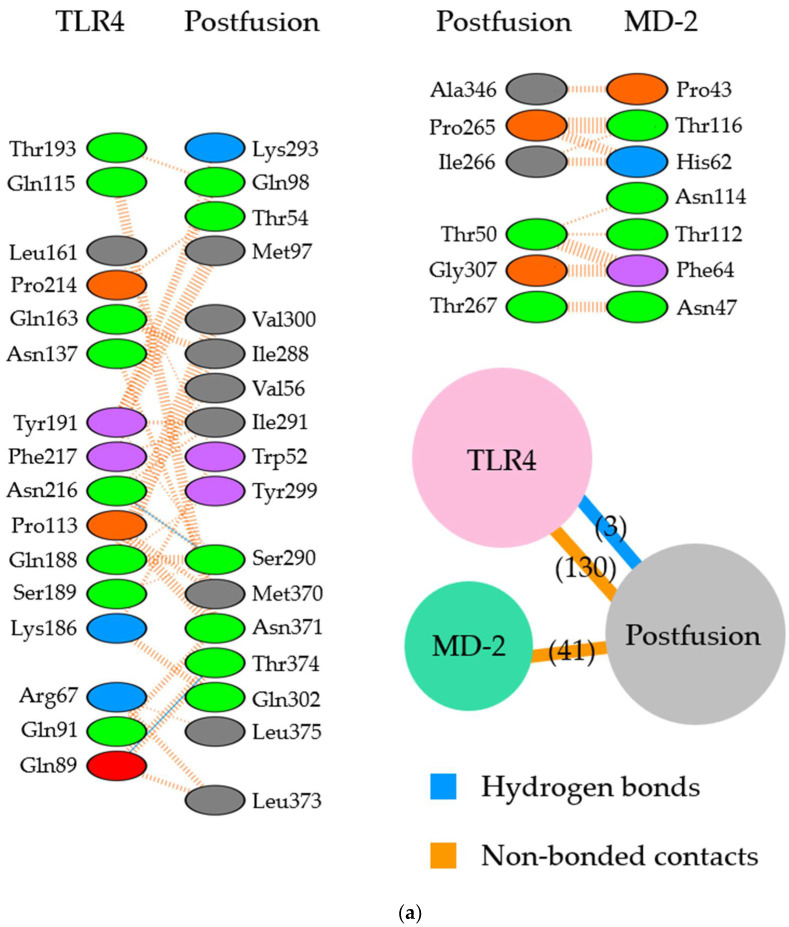

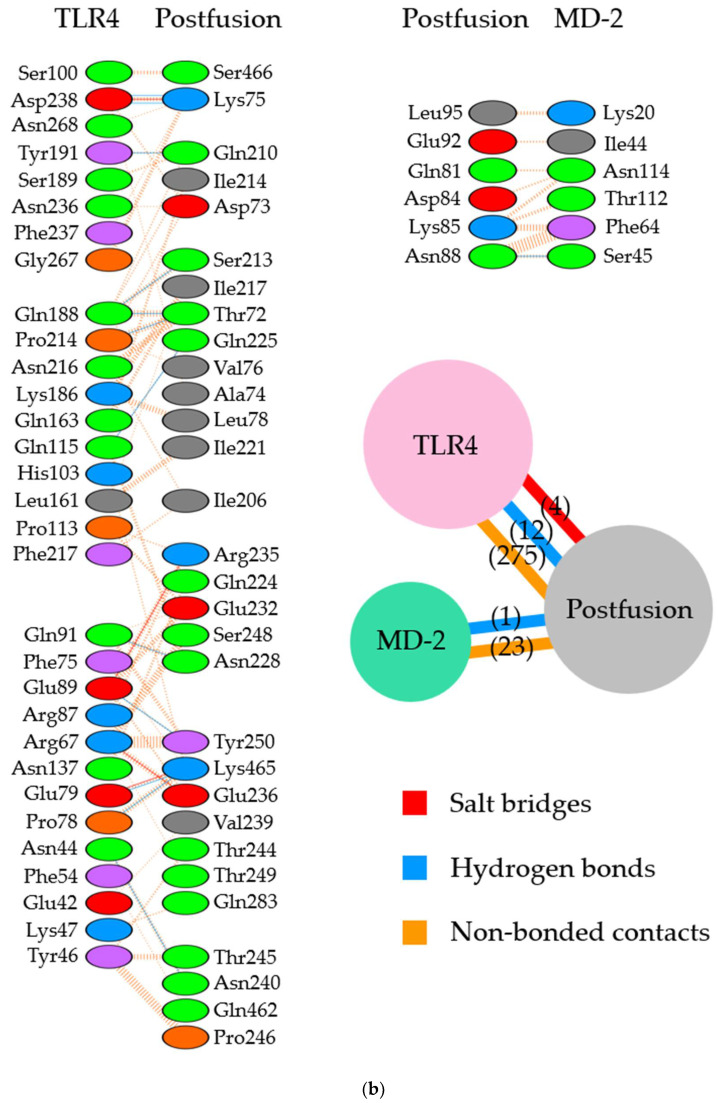
Illustration of the molecular interactions between respiratory syncytial virus (RSV) fusion proteins and Toll-like receptor 4/myeloid differentiation factor-2 (TLR4/MD-2). (**a**) The molecular interactions between RSV postfusion proteins in which site II was designated as the binding site and TLR4/MD-2 complexes. (**b**) The molecular interactions between RSV postfusion proteins in which site IV was specified as the binding site and TLR4/MD-2 complexes. The molecular interactions are depicted as follows: salt bridge (red), hydrogen bonds (blue), and non-bonded contacts (orange). The number on lines represents the number of bonds in the 2D protein diagram.

We analyzed the detailed molecular interactions between the F proteins (prefusion/postfusion type) and TLR4/MD-2 complex in each suitably selected model. As shown in Figure 2, Figure 3, Figure 4 and Figure 5 both sites II and IV in the prefusion proteins were involved in these interactions.

In the site II-associated prefusion protein/TLR4/MD-2 complex model, the interactions between the prefusion proteins and TLR4 were mediated by eight hydrogen bonds (two hydrogen bonds between Gln361 and Ser521), two salt bridges, and 262 non-bonded contacts, whereas the MD-2 molecules did not interact with the prefusion proteins (Figure 2a and Figure 3a). Lys272 and Asn276 at site II of the prefusion proteins formed hydrogen bonds with Lys541, Arg496, Asn517, and Ser520 in the TLR4 molecule (Table 2). There was no salt bridge between site II of the prefusion proteins and the TLR4 molecule. The intermolecular distances between site II of the prefusion proteins and TLR4 were 2.80–3.06Å (Table 2). In the docking complex model in which site II was designated as the binding site, the binding affinity between prefusion proteins and the TLR4/MD-2 complexes was calculated to be −8.3 kcal/mol (Table 3). The number of ICs between site II of the prefusion proteins and the TLR4/MD-2 complex was as follows: charged/charged, 14; charged/apolar, 28; polar/polar, 33; and polar/apolar, 20. In this model, the %NIC values of the apolar and charged were 34.42% and 24.39%, respectively.

Next, in the site IV-associated prefusion protein/TLR4/MD-2 complex model, the prefusion protein interacted with TLR4 through five hydrogen bonds, no salt bridge, and 197 non-bonded contacts and with MD-2 through 21 non-bonded interactions (Figure 2b and Figure 3b). At site IV of the prefusion proteins, there were no hydrogen bond non-salt bridges connected to the TLR4 molecule. In this docking complex model, the predicted binding affinity between prefusion proteins and TLR4/MD-2 complexes was −12.9 kcal/mol (Table 3). In the docking complex models in which site IV was designated as the binding site, the number of ICs between the prefusion proteins and TLR4/MD-2 was as follows: charged/charged, 7; charged/apolar, 29; polar/polar, 13; and polar/apolar, 25. The %NIC values for apolar and charged elements were 33.91% and 24.68%, respectively. In addition, the 3D structural models and intermolecular interactions between the TLR4/MD-2 complex and the prefusion protein in the best-scoring models of other antigenic sites (Ø, I, III, and V) are shown in Figures S1 and S2.

### 3.3. Molecular Interactions between Postfusion Proteins and TLR4/MD-2

The molecular interactions between the postfusion proteins and TLR4/MD-2 were analyzed (Figure 4). In the site II-associated postfusion protein/TLR4/MD-2 complex model, the postfusion proteins interacted with TLR4 mediated by three hydrogen bonds (two hydrogen bonds between Thr374 and Glu89), no salt bridge, and 130 non-bonded contacts and with MD-2 through 41 non-bonded contacts (Figure 4a and Figure 5a). No interaction by hydrogen bonds, salt bridges, or non-bonded contacts existed between the postfusion protein site II and the TLR4 molecule. There were 23 non-bonded contacts between the postfusion protein site IV and MD-2 molecules. In this docking complex model, the estimated binding affinity between the post-fusion proteins and TLR4/MD-2 was −15.4 kcal/mol (Table 3). The results indicated that the molecular affinity between the postfusion proteins and TLR4/MD-2 complexes was considerably higher than that between the prefusion proteins and TLR4/MD-2 complexes in the docking complex model in which site II was designated as the binding site. The ICs found between the postfusion proteins and TLR4/MD-2 complexes in this model were as follows: charged/charged, 1; charged/apolar, 14; polar/polar, 14; and polar/apolar, 46. The percentages of apolar and charged NICs in this model were 34.48% and 23.69%, respectively.

Subsequently, in the site IV-associated postfusion protein/TLR4/MD-2 complex model, the postfusion proteins interacted with TLR4 via 12 hydrogen bonds (two hydrogen bonds between Lys75 and Asp238), 4 salt bridges, and 275 non-bonded contacts and with MD-2 through 23 non-bonded contacts and one hydrogen bond (Figure 4b and Figure 5b). Lys465 in site IV of postfusion proteins interacted with Glu79 in TLR4 salt bridges. Likewise, Lys465 at site IV of postfusion proteins hydrogen-bonded to Pro78 and Glu79 in the TLR4 molecules. The calculated distance between the postfusion proteins site IV and TLR4/MD-2 complexes was between 2.51 and 3.13 Å. (Table 4). In this docking model, the binding affinity between the postfusion proteins and TLR4/MD-2 was −14.3 kcal/mol (Table 3). The results revealed that the molecular affinity between the postfusion proteins and TLR4/MD-2 complexes was increased in the docking complex model in which site IV was designated as the binding site. In this model, postfusion proteins and TLR4/MD-2 complexes had 14 charged/charged, 40 charged/apolar, 21 polar/polar, and 30 polar/apolar ICs. The percentage of apolar and charged NICs was 34.38% and 23.52%, respectively.

### 3.4. Molecular Docking between TLR4/MD-2 and LPS, and RSV Prefusion Protein and Antibody CR9501

Molecular docking was performed between TLR4/MD-2 and LPS to compare it with an experimentally determined 3D structure (Figure S3). As shown in Table S1, 41 out of 57 molecular interactions and interacting sites in the TLR4/MD-2/LPS complex docking model were consistent with those in the TLR4/MD-2/LPS complex structure determined by X-ray crystallography. Similarly, a docking simulation was conducted between the RSV prefusion protein and antibody CR9501 (Figure S4). Table S2 shows that 49 out of 75 molecular interactions and interacting sites in the prefusion protein/antibody complex docking model correspond to those in the cryo-EM structure of the prefusion protein/antibody complex.

### 3.5. Comparison of Interacting Sites between the Present Docking Models and Prediction by ScanNet

ScanNet was used to predict the binding probability of all residues in the prefusion and postfusion proteins. Among the 449 residues in the prefusion protein, the sites with a binding probability > 0.5 were 64 residues (14.3%) (Table S3). In addition, among the 441 residues in the postfusion protein, the sites with a binding probability > 0.5 were 153 residues (34.7%) (Table S3). In the site II-associated F protein/TLR4/MD-2 complex docking model, the number of interacting sites corresponding to sites with a binding probability > 0.5 was five out of 34 interacting sites (14.7%) in the prefusion protein and 10 out of 26 interacting sites (38.5%) in the postfusion protein. In the site IV-associated F protein/TLR4/MD-2 complex docking model, the number of interacting sites corresponding to sites with a binding probability > 0.5 was 11 out of 33 interacting sites (33.3%) in the prefusion protein and 25 out of 50 interacting sites (50.0%) in the postfusion protein.

## 4. Discussion

The detailed molecular interactions among RSV prefusion proteins, postfusion proteins, and the host cellular receptor TLR4/MD-2 complex were analyzed using authentic bioinformatics technologies. First, both sites II and IV of the prefusion and postfusion proteins interacted with the TLR4/MD-2 complex. The NT-Ab binding sites of sites II and IV of the proteins (prefusion and postfusion) were fully involved in the interactions between these proteins and the TLR4 molecule. Second, the binding affinity between postfusion proteins and the TLR4/MD-2 complexes was increased compared to that of prefusion proteins and the TLR4/MD-2 complexes. To the best of our knowledge, a previous report suggested that the host cellular receptors against the RSV fusion protein were TLR4 molecules using only cell biological and immunological methods [18]. Therefore, the detailed molecular interactions among RSV prefusion proteins, postfusion proteins, and the TLR4/MD-2 complexes using in silico technologies in this study may be the first.

Previous reports suggested that distinct antigenicity of RSV was found between prefusion and postfusion proteins [12]. Moreover, prefusion proteins may be associated with the RSV infection to the host cells, but postfusion proteins may not be associated [12]. Indeed, a representative NT-Ab agent, such as palivizumab, can bind to the NT-Ab binding sites (Site II) of prefusion proteins, leading to the prevention of RSV infection to host cells [32]. However, the molecular interactions based on detailed 3D structures among prefusion proteins, postfusion proteins, and the TLR4/MD-2 complexes may be little understood. The present simulation data clearly showed that the NT-Ab binding sites of prefusion and postfusion proteins were completely involved in F proteins and ligands of TLR4/MD-2 complexes (Figure 2, Figure 3, Figure 4 and Figure 5). These compatibilities between the NT-Ab binding sites of virus proteins and cellular receptor binding sites (TLR4/MD-2 complexes) may explain why palivizumab prevents the infection to host cells.

In this study, both models, in which sites II and IV were designated as the binding sites, showed an increasing binding affinity for the TLR4/MD-2 complex as prefusion changed to postfusion. Moreover, the selected docking models at sites II and IV differed from each other. This may indicate that binding to the TLR4/MD-2 complex occurs independently at sites II and IV of RSV. Furthermore, the increased binding affinity appears to be consistent with binding to TLR4, triggering the change from prefusion to postfusion in the F protein. These results suggest that conformational changes in the F protein promote viral adsorption to the host cell. However, the mechanism by which F proteins change from prefusion to postfusion after binding of TLR4 to F proteins may need to be elucidated for further studies.

The binding sites of the TLR4/MD-2 complex for binding to the F protein differed from those for binding to LPS (Table S1). TLR4 is one of the leading receptors for innate immune responses, and LPS is a well-known agonist of TLR4. A previous report showed that the LPS-binding site for the TLR4/MD-2 complex is located mainly on MD-2 [33]. In contrast, in the present study, the F-proteins bound mainly to TLR4 in the TLR4/MD-2 complex. This suggests that RSV entry into cells does not necessarily promote an innate immune response to TLR4, although the relationships between the active sites of TLR4 and the F protein may not be known.

In general, cell membranes composed of phospholipid bilayers have strongly similar polarity [34]. Therefore, cell membrane fusion may hard syncytiumly occur due to electric repulsion with each other [35]. Previous reports showed that postfusion proteins are associated with syncytium formation due to cell membrane fusion in RSV-infected cells [8]. Although statistical analysis of the binding affinity was not available due to the method of docking simulation, drastic changes in binding affinity were observed due to conformational changes between prefusion and postfusion proteins. In this study, the binding affinity was calculated based on the equation involved in the HADDOCK web server (Equation (1)). The present data showed that the number of polar/polar ICs were drastically decreased in inter-residue contacts between postfusion proteins and the TLR4/MD-2 complexes compared to those in inter-residue contacts between prefusion proteins and the TLR4/MD-2 complexes in the site II-associated F protein/TLR4/MD-2 complex model (Table 3). In contrast, at site IV, there was an increase in the number of polar/polar ICs in inter-residue contacts between postfusion proteins and the TLR4/MD-2 complexes compared to those in inter-residue contacts between prefusion proteins and the TLR4/MD-2 complexes (Table 3). However, at the same time, the number of charged/charged, charged/apolar, and polar/apolar IC complexes were increased in inter-residue contacts between postfusion proteins and the TLR4/MD-2 complexes compared to those in inter-residue contacts between prefusion proteins and the TLR4/MD-2 complexes in the site IV-associated F protein/TLR4/MD-2 complex model (Table 3). These changes affected the molecular affinity values, although, the effect of these differences in changing the manner of inter-residue contacts between sites II and IV is not known. These binding affinity changes may contribute to syncytium formation in virus-infected cells, including RSV, measles virus, and mumps virus, although further in silico studies may be needed for other viruses with fusion proteins.

The docking models generated by HDOCK were rescored to obtain suitable simulation data. The scoring function, which plunks near-native solutions from thousands of possible solutions created by docking algorithms, plays a pivotal role in the reliability of docking simulation [36]. Hence, combining the results from multiple distinct scoring functions may allow us to obtain better solutions than using a single scoring function [37]. The scoring functions can be roughly divided into four categories: force-field-based, empirical, knowledge-based, and machine-learning-based [38]. HDOCK, HawkDock, and PPI-Affinity use knowledge, force field, and machine learning-based scoring functions, respectively [24,25,26]. Thus, in addition to HDOCK, HawkDock and PPI-Affinity were used to determine the optimal docking model.

Next, HDOCK data were validated by redocking and binding site predictions. HDOCK employs knowledge-based scoring functions, which can provide discrepancies in the reliability of results depending on a given protein due to limited training sets of crystal structures [24,36,39]. Thus, a validation approach was also performed on the molecular interactions between the TLR4/MD-2 complexes and LPS, and the RSV prefusion protein and antibody in the present study (Tables S1 and S2). Consequently, the present simulation data are compatible with the experimentally determined structure [33,40]. Thus, our simulation data may be relevant to native structures, although other physicochemical analyses, such as X-ray diffraction analysis, were not performed. Furthermore, we validated the interacting sites in the docking models by using ScanNet. In the site IV-associated F protein/TLR4/MD-2 complex model, the percentage correspondence between the interacting sites of the docking model and sites for which a high binding probability was predicted by ScanNet was relatively high, whereas that in the site II-associated F protein/TLR4/MD-2 complex model was not as high. Although these validation approaches could increase the reliability of the docking simulation, additional analysis of the F protein/TLR4/MD2 complex structure may refine the present findings. Molecular dynamics simulation may be a powerful tool for obtaining a near-native docking model. However, this may be difficult to perform in authentic bioinformatic laboratories. This could be an issue for future research.

Finally, docking models in which TLR4/MD2 bound to sites II and IV of the F protein were selected for the present study. In contrast, based on the binding site probability prediction and the ranking of docking models by multiple scoring functions, it is possible that TLR4 binds more readily to site IV in the F protein. However, elucidation of this through in silico methodologies is limited; interdisciplinary approaches, including in vitro and in vivo, may be needed for future studies.

## 5. Conclusions

We studied the detailed molecular interactions between RSV F proteins and TLR4/MD-2 complexes in silico. The NT-Ab binding sites in both prefusion and postfusion proteins at sites II and IV may be involved in counter ligands as the TLR4/MD-2 complexes. Furthermore, the present simulation models suggested that F proteins could strongly bind to the TLR4/MD-2 complexes due to conformational changes from prefusion to postfusion proteins, increasing the binding affinity. These new findings may contribute to better understand the infectivity and pathogenicity (syncytium formation) of RSV infection.

## Figures and Tables

**Table 1:** (a). Top five docking models of prefusion proteins based on HDOCK, HawkDock, and PPI-Affinity scores. (b). Top five docking models of postfusion proteins based on HDOCK, HawkDock, and PPI-Affinity scores.

(a)				
**Ranking**	**HDOCK**	**HawkDock**	**PPI-Affinity**	**Antigenic Sites**
1st	2	8	10	Site IV
2nd	12	15	7	
3rd	1	27	9	Site II
3rd	15	12	10	
5th	25	3	11	
(b)				
**Ranking**	**HDOCK**	**HawkDock**	**PPI-Affinity**	**Antigenic Sites**
1st	1	3	3	Site II
2nd	2	4	2	
3rd	5	2	3	
4th	4	6	4	
4th	6	7	1	
1st	1	3	3	Site IV
2nd	6	5	1	
3rd	2	9	2	
4th	5	7	3	
5th	11	2	3	

**Table 2:** Key interactions, interacting residues, and intermolecular distances between the prefusion proteins site II and TLR4 molecule.

Interaction Type	Fusion Protein	TLR4	Distance (Å)
Hydrogen bonds	Lys272	Lys541	2.81
	Asn276	Arg496	2.80
	Asn276	Asn517	3.06
	Asn276	Ser520	2.98

TLR4, Toll-like receptor 4.

**Table 3:** Number of ICs and %NIS, and binding affinity estimated by HADDOCK.

Fusion Protein	Prefusion		Postfusion	
Antigenic Sites	Site II	Site IV	Site II	Site IV
ICs charged/charged (no.)	14	7	1	14
ICs charged/apolar (no.)	28	29	14	40
ICs polar/polar (no.)	33	13	14	21
ICs polar/apolar (no.)	20	25	46	30
%NIS apolar (%)	34.42	33.91	34.48	34.38
%NIS charged (%)	24.39	24.68	23.69	23.52
Binding Affinity (kcal/mol)	−8.3	−12.9	−15.4	−14.3

ICs, Interfacial contacts; NIS, Non-interaction surfaces.

**Table 4:** Key interactions, interacting residues, and intermolecular distances between the postfusion protein site IV and TLR4 molecule.

Interaction Type	Fusion Protein	TLR4	Distance (Å)
Hydrogen bonds	Lys465	Pro78	3.13
	Lys465	Glu79	2.51
Salt bridges	Lys465	Glu79	2.51

TLR4, Toll-like receptor 4.

## Data Availability

The datasets generated and/or analyzed during the current study are available from the corresponding author on reasonable request.

## Supplementary Materials

The following supporting information can be downloaded at: https://www.mdpi.com/article/10.3390/v14112382/s1, Figure S1. Diagram of binding conformations between respiratory syncytial virus (RSV) prefusion proteins and Toll-like receptor 4/myeloid differentiation factor-2 (TLR4/MD-2).; Figure S2. Illustration of the molecular interactions between respiratory syncytial virus (RSV) fusion proteins and Toll-like receptor 4/myeloid differentiation factor-2 (TLR4/MD-2).; Figure S3: Diagram illustrating the comparison of the Toll-like receptor 4/myeloid differentiation factor-2/lipopolysaccharide (TLR4/MD-2/LPS) complex structures with HDOCK and X-ray crystallography data.; Figure S4: Diagram illustrating the comparison of the respiratory syncytial virus (RSV) prefusion protein/antibody CR9501 complex between the HDOCK and Cryo-electron microscopy (cryo-EM) structure data.; Table S1a: Consistent molecular interactions and interacting sites of TLR4/MD-2/LPS complex in the HDOCK and X-ray crystallography data.; Table S1b: Inconsistent molecular interactions and interacting sites of TLR4/MD-2/LPS complex in the HDOCK and X-ray crystallography data.; Table S2a: Consistent molecular interactions and interacting sites of respiratory syncytial virus (RSV) prefusion protein/antibody CR9501 complex in the HDOCK and Cryo-electron microscopy (cryo-EM) structure data.; Table S2b: Inconsistent molecular interactions and interacting sites of respiratory syncytial virus (RSV) prefusion protein/antibody CR9501 complex in the HDOCK and Cryo-electron microscopy (cryo-EM) structure data.; Table S3: Sites with a binding probability > 0.5 as predicted by ScanNet in the prefusion and postfusion proteins.
