# Supplementary material for: Detailed Molecular Interactions between Respiratory Syncytial Virus Fusion Protein and the TLR4/MD-2 Complex In Silico
Source: Viruses. 2022 Oct 28;14(11):2382. doi: 10.3390/v14112382 (PMC9694959; doi:10.3390/v14112382)
Supplement: Supplementary file 1 [file viruses-14-02382-s001.zip › Supplemental Figure S1.pdf]

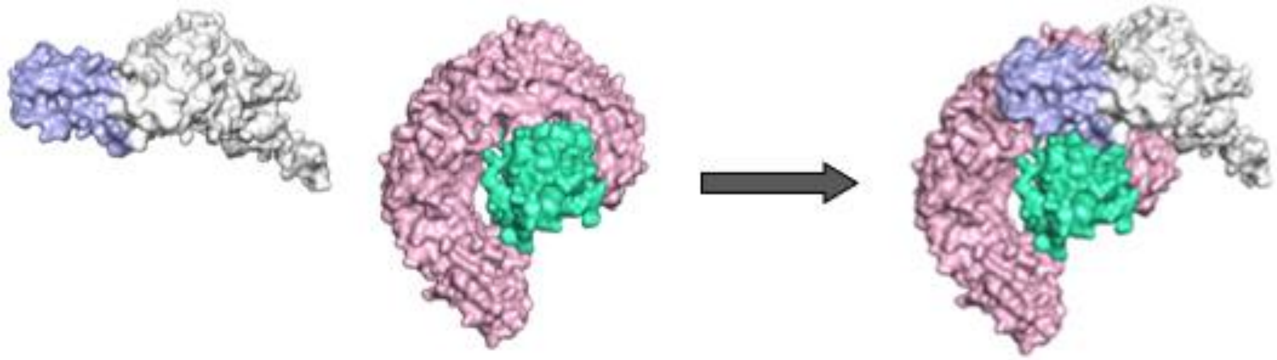

(a)

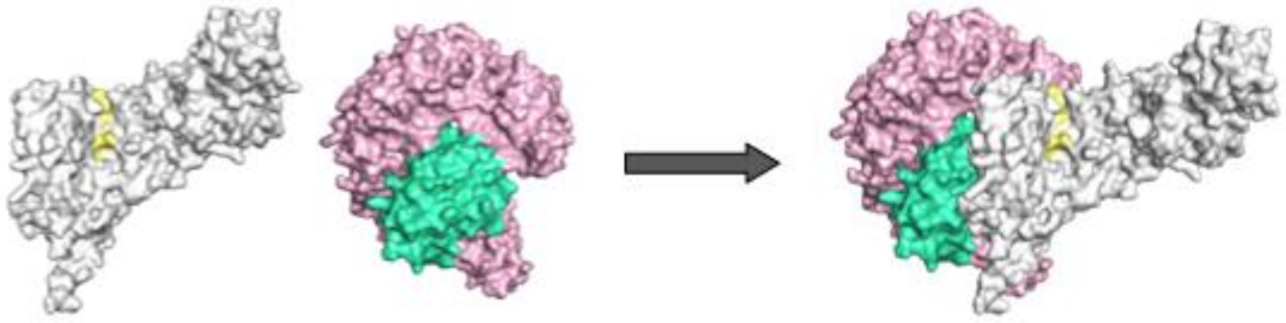

(b)

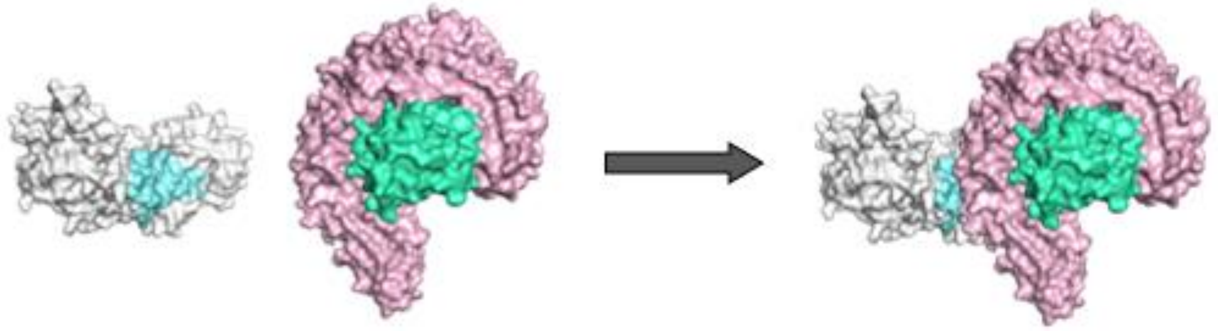

(c)

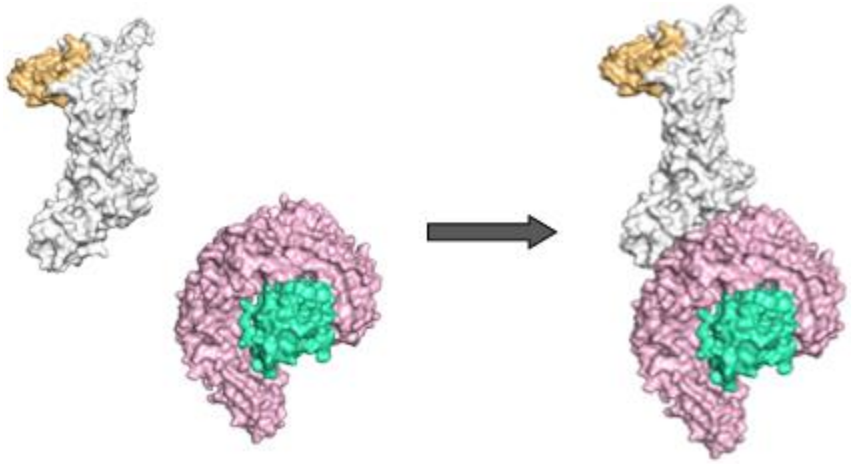

(d)

Figure S1. Diagram of binding conformations between respiratory syncytial virus (RSV) prefusion proteins and Toll-like receptor 4/myeloid differentiation factor-2 (TLR4/MD-2). (a) Three-dimensional (3D) structures of RSV prefusion proteins, TLR4/MD-2 complexes, and the protein-protein docking models in which site Ø was designated as the binding site. (b) 3D structures of RSV prefusion proteins, TLR4/MD-2 complexes, and the protein-protein docking models in which site I was specified as the binding site. (c) 3D structures of RSV prefusion proteins, TLR4/MD-2 complexes, and the protein-protein docking models in which site III was specified as the binding site. (d) 3D structures of RSV prefusion proteins, TLR4/MD-2 complexes, and the protein-protein docking models in which site V was specified as the binding site. Sites Ø, I, III, and V in RSV fusion proteins are shown in light purple, light yellow, light blue, and light orange, respectively. The TLR4 and MD-2 are colored pink and light green, respectively.
