# Supplementary material for: Detailed Molecular Interactions between Respiratory Syncytial Virus Fusion Protein and the TLR4/MD-2 Complex In Silico
Source: Viruses. 2022 Oct 28;14(11):2382. doi: 10.3390/v14112382 (PMC9694959; doi:10.3390/v14112382)
Supplement: Supplementary file 1 [file viruses-14-02382-s001.zip › Supplemental Figure S2.pdf]

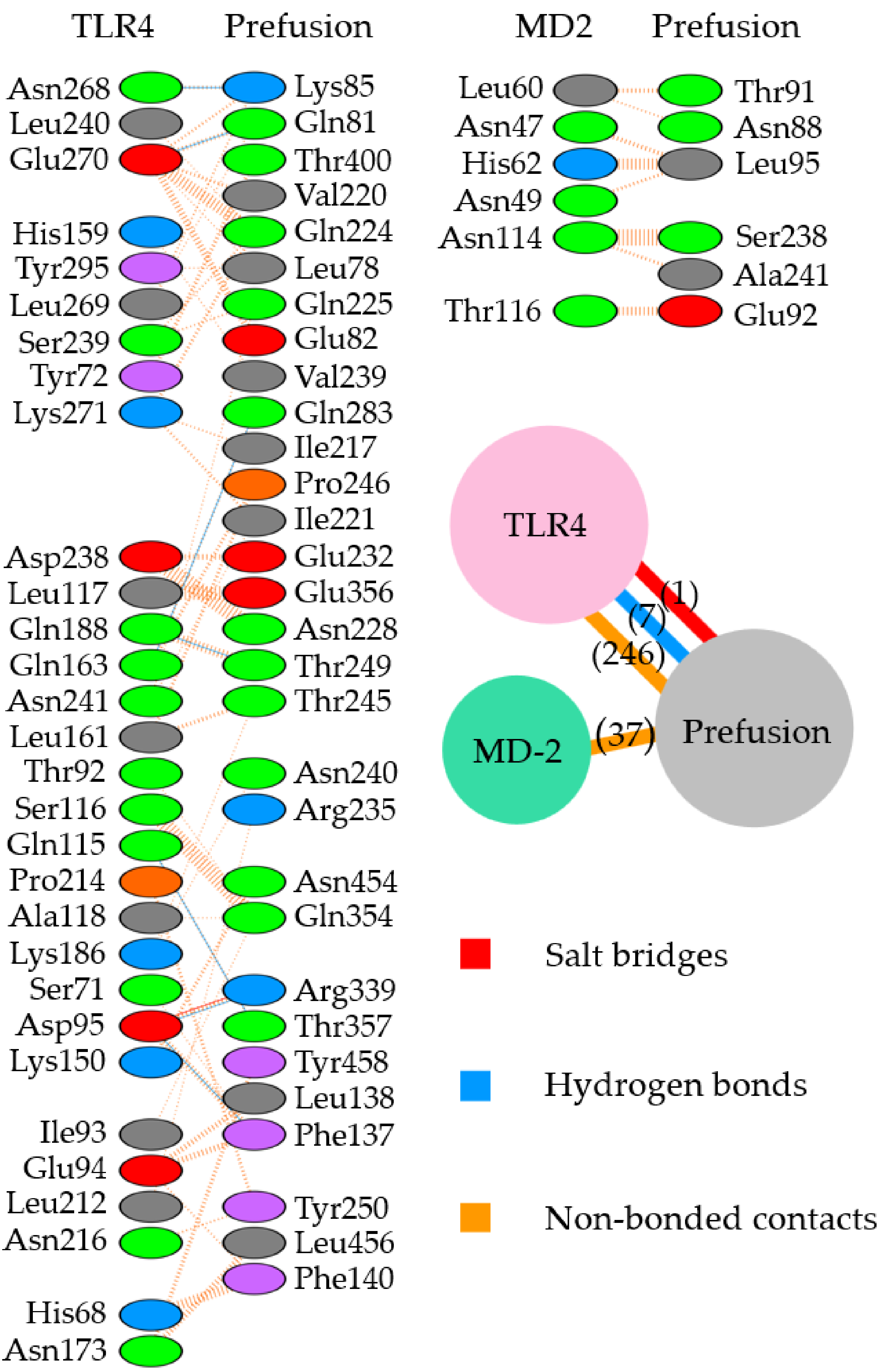
TLR4
Prefusion
MD2
Prefusion
Asn268
Leu240
Glu270
His159
Tyr295
Leu269
Ser239
Tyr72
Lys271
Asp238
Leu117
Gln188
Gln163
Asn241
Leu161
Thr92
Ser116
Gln115
Pro214
Ala118
Lys186
Ser71
Asp95
Lys150
Ile93
Glu94
Leu212
Asn216
His68
Asn173
Lys85
Gln81
Thr400
Val220
Gln224
Leu78
Gln225
Glu82
Val239
Gln283
Ile217
Pro246
Ile221
Glu232
Glu356
Asn228
Thr249
Thr245
Asn240
Arg235
Asn454
Gln354
Arg339
Thr357
Tyr458
Leu138
Phe137
Tyr250
Leu456
Phe140
Leu60
Asn47
His62
Asn49
Asn114
Thr116
Thr91
Asn88
Leu95
Ser238
Ala241
Glu92
TLR4
(1)
(7)
(246)
MD-2
(37)
Prefusion
Salt bridges
Hydrogen bonds
Non-bonded contacts

(a)

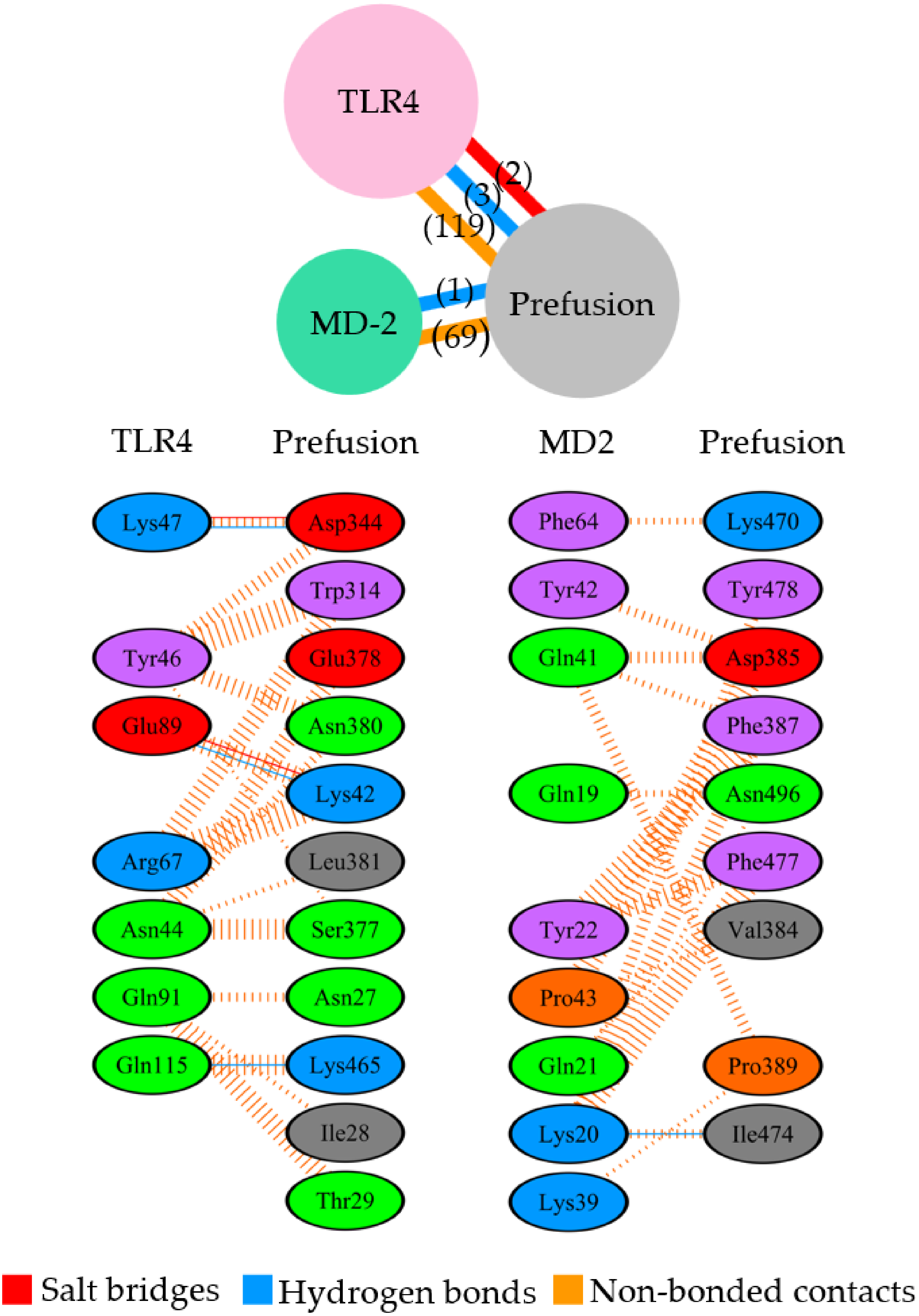

TLR4
Prefusion
MD-2
(2)
(3)
(119)
(1)
(69)
TLR4
Prefusion
MD2
Prefusion
Lys47
Asp344
Trp314
Tyr46
Glu378
Glu89
Asn380
Lys42
Arg67
Leu381
Asn44
Ser377
Gln91
Asn27
Gln115
Lys465
Ile28
Thr29
Phe64
Lys470
Tyr42
Tyr478
Gln41
Asp385
Phe387
Gln19
Asn496
Phe477
Tyr22
Val384
Pro43
Gln21
Pro389
Lys20
Ile474
Lys39
Salt bridges
Hydrogen bonds
Non-bonded contacts

(b)

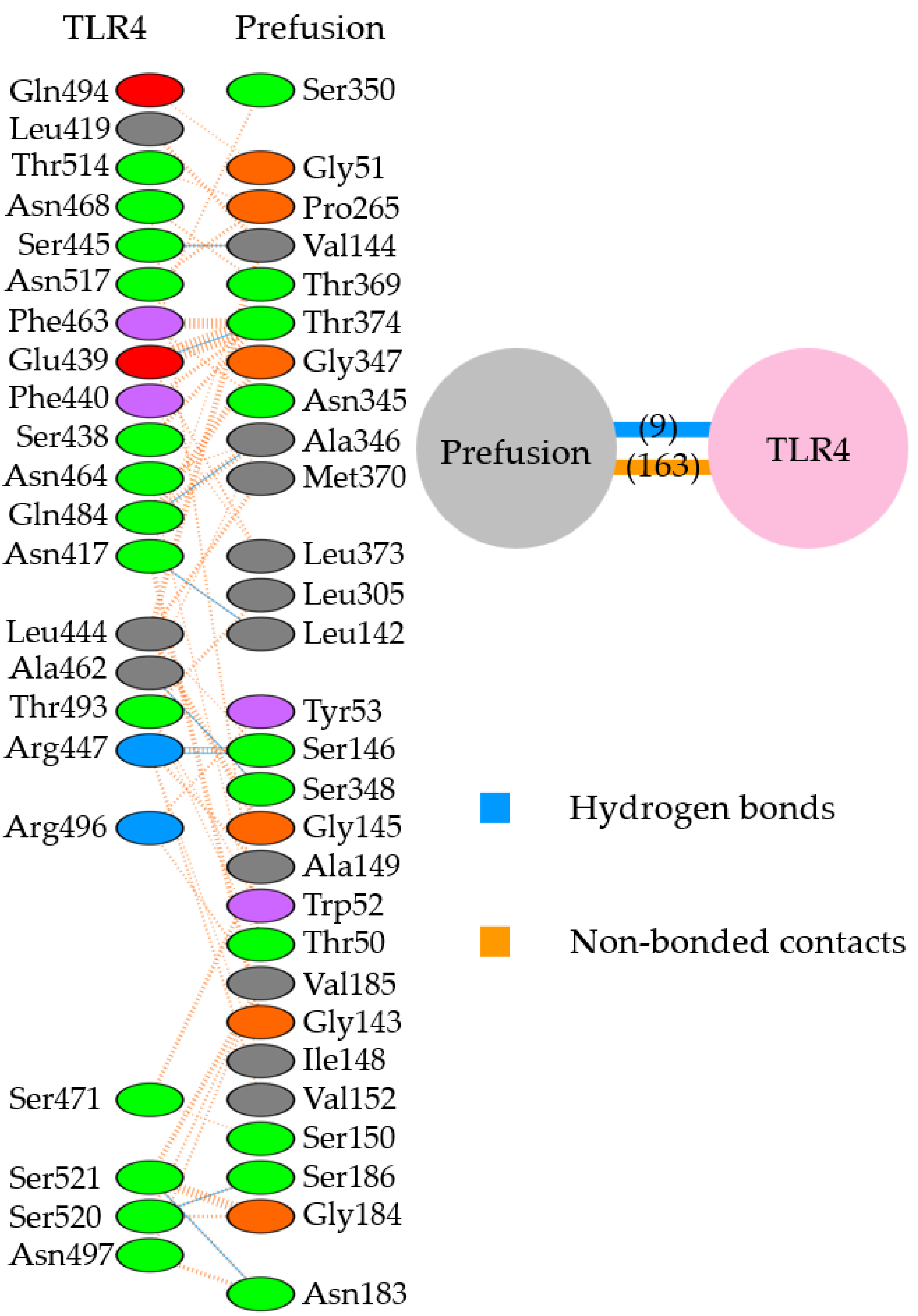
TLR4
Prefusion
Gln494
Leu419
Thr514
Asn468
Ser445
Asn517
Phe463
Glu439
Phe440
Ser438
Asn464
Gln484
Asn417
Leu444
Ala462
Thr493
Arg447
Arg496
Ser471
Ser521
Ser520
Asn497
Ser350
Gly51
Pro265
Val144
Thr369
Thr374
Gly347
Asn345
Ala346
Met370
Leu373
Leu305
Leu142
Tyr53
Ser146
Ser348
Gly145
Ala149
Trp52
Thr50
Val185
Gly143
Ile148
Val152
Ser150
Ser186
Gly184
Asn183
Prefusion
(9)
(163)
TLR4
Hydrogen bonds
Non-bonded contacts

(c)

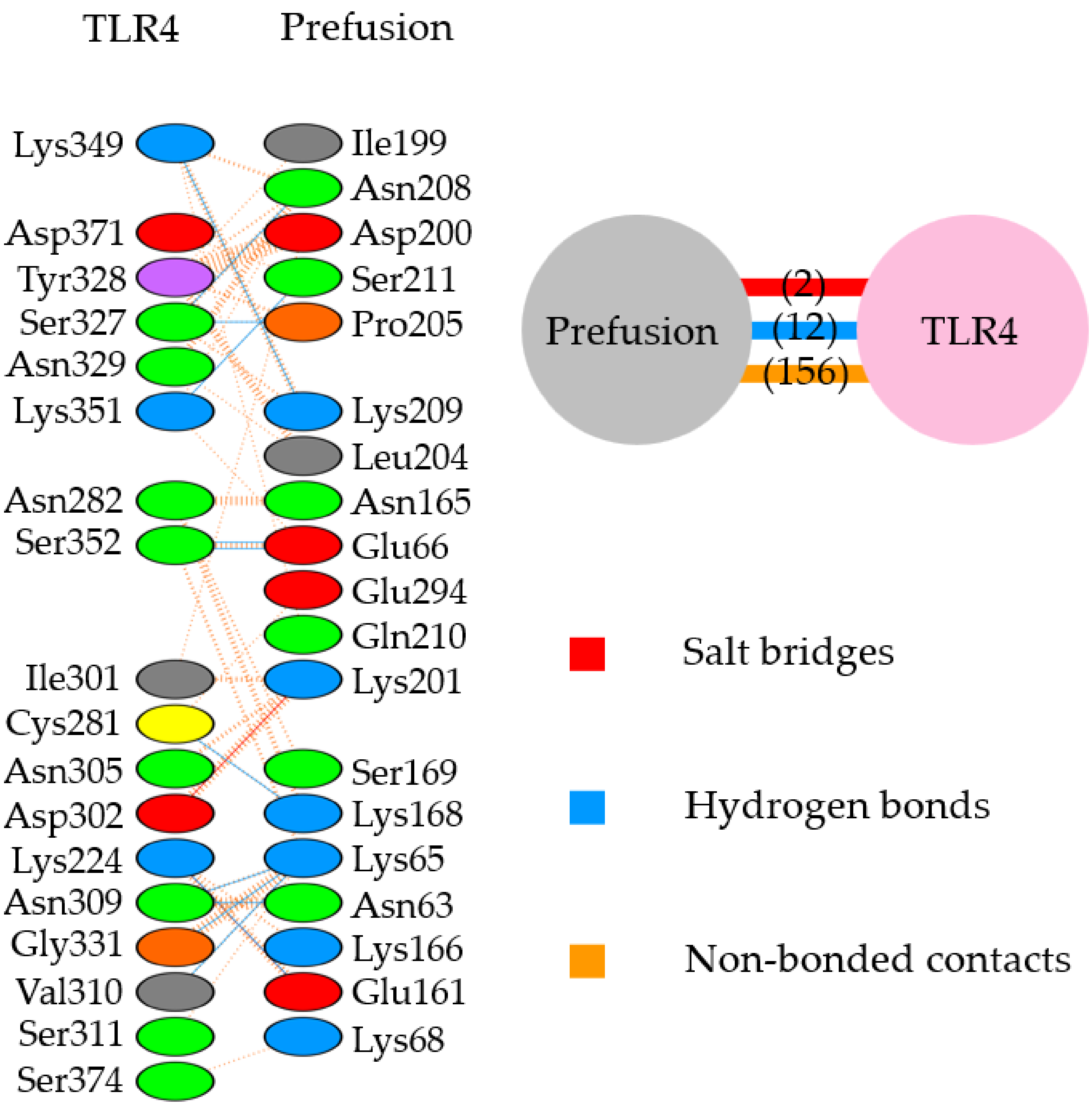

(d)

Figure S2. Illustration of the molecular interactions between respiratory syncytial virus (RSV) fusion proteins and Toll-like receptor 4/myeloid differentiation factor-2 (TLR4/MD-2). (a) The molecular interactions between RSV prefusion proteins in which site Ø was designated as the binding site and TLR4/MD-2 complexes. (b) The molecular interactions between RSV prefusion proteins in which site I was specified as the binding site and TLR4/MD-2 complexes. (c) The molecular interactions between RSV prefusion proteins in which site III was specified as the binding site and TLR4/MD-2 complexes. (d) The molecular interactions between RSV prefusion proteins in which site V was specified as the binding site and TLR4/MD-2 complexes. The molecular interactions are depicted as follows: salt bridge (red), hydrogen bonds (blue), and nonbonded contacts (orange). The number on lines represents the number of bonds in the 2D protein diagram.
