# Supplementary material for: Detailed Molecular Interactions between Respiratory Syncytial Virus Fusion Protein and the TLR4/MD-2 Complex In Silico
Source: Viruses. 2022 Oct 28;14(11):2382. doi: 10.3390/v14112382 (PMC9694959; doi:10.3390/v14112382)
Supplement: Supplementary file 1 [file viruses-14-02382-s001.zip › Supplemental Figure S3.pdf]

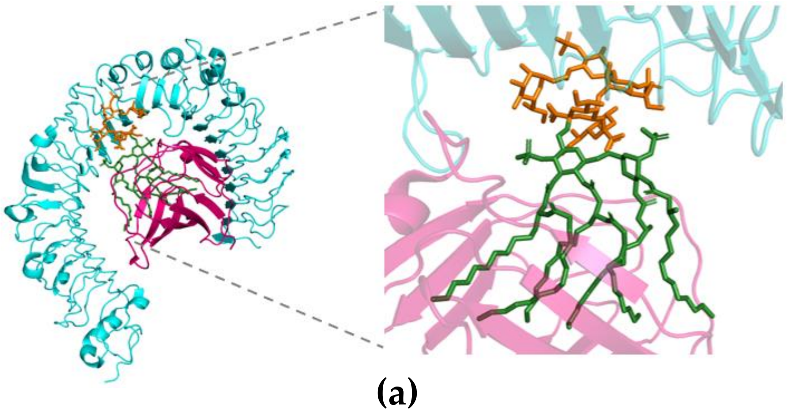

(a)

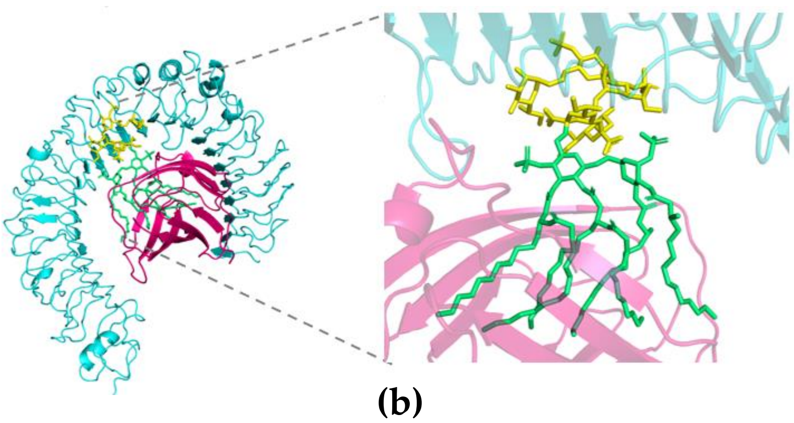

(b)

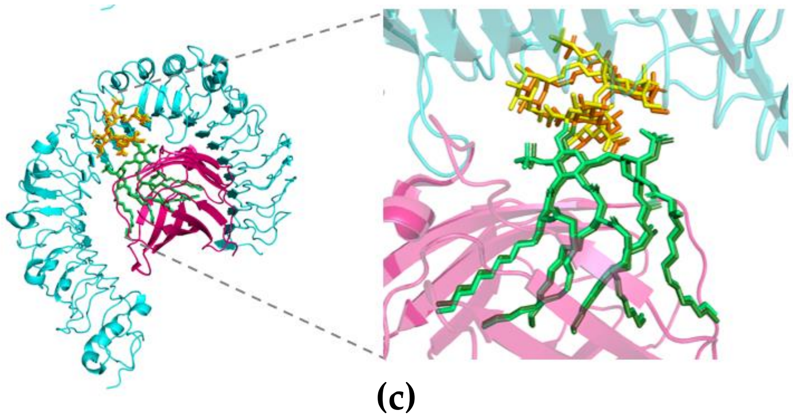

(c)

Figure S3. Diagram illustrating the comparison of the Toll-like receptor 4/myeloid differentiation factor-2/lipopolysaccharide (TLR4/MD-2/LPS) complex structures with HDOCK and X-ray crystallography data. The three-dimensional (3D) structures of TLR4 and MD-2 are presented in a cartoon style, whereas LPS (core oligosaccharide and lipid A) is depicted in stick representation. TLR4 and MD-2 are shown in light blue and dark pink, respectively. (a) 3D structures of the TLR4/MD-2/LPS complexes determined by X-ray crystallography. The core oligosaccharide and lipid A of LPS are shown in orange and dark green, respectively. (b) 3D structure of the TLR4/MD-2/LPS complexes of HDCOK data. The core oligosaccharide and lipid A of LPS are shown in yellow and light green, respectively. (c) Superimposition 3D model of the TLR4/MD-2/LPS complexes of HDOCK and X-ray crystallography data.
