# Supplementary material for: Detailed Molecular Interactions between Respiratory Syncytial Virus Fusion Protein and the TLR4/MD-2 Complex In Silico
Source: Viruses. 2022 Oct 28;14(11):2382. doi: 10.3390/v14112382 (PMC9694959; doi:10.3390/v14112382)
Supplement: Supplementary file 1 [file viruses-14-02382-s001.zip › Supplemental Figure S4.pdf]

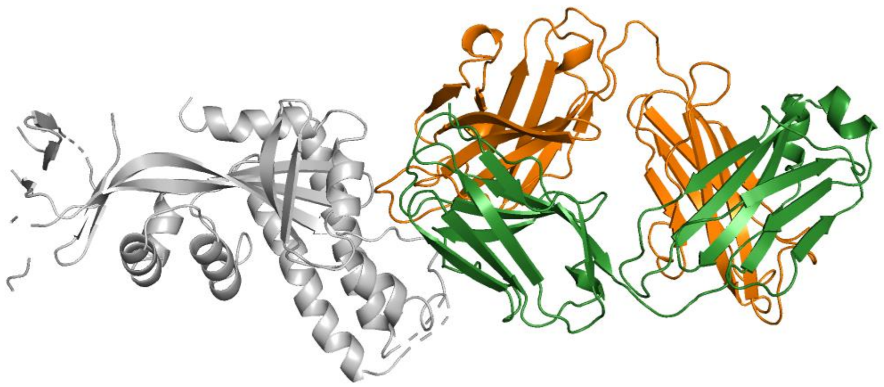

(a)

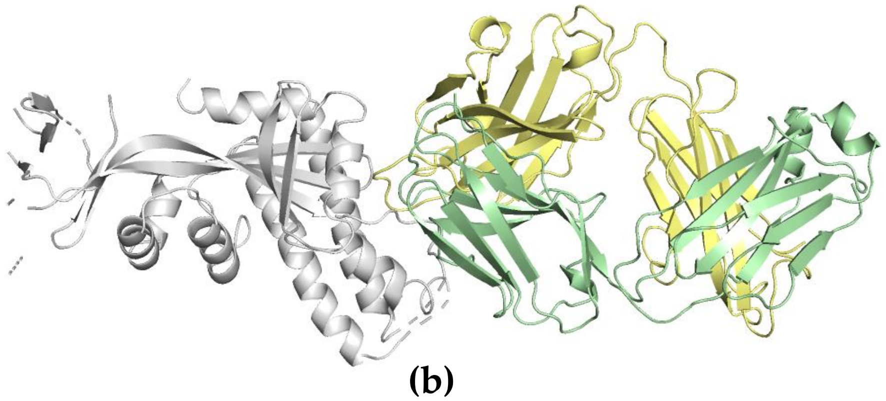

(b)

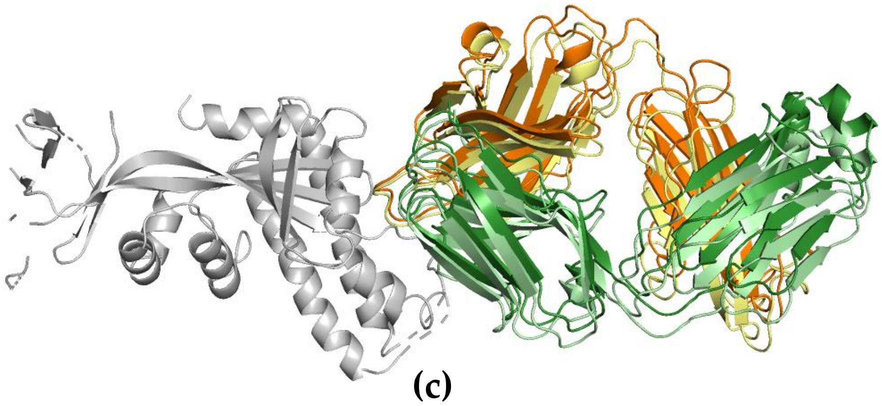

(c)

Figure S4. Diagram illustrating the comparison of the Respiratory Syncytial Virus (RSV) prefusion protein/antibody CR9501 complex between the HDOCK and Cryo-electron microscopy (cryo-EM) structure data. The three-dimensional (3D) structures of RSV prefusion protein and antibody CR9501 are presented in a cartoon style. RSV prefusion protein is shown in gray. (a) 3D structures of the RSV prefusion protein/antibody CR9501 complexes determined by cryo-EM structure. Fragment antigen-binding region (Fab) heavy chain and light chain of antibody CR9501 are shown in orange and dark green, respectively. (b) 3D structure of the RSV prefusion protein/antibody CR9501 complexes of HDCOK data. Fab heavy chain and light chain of antibody CR9501 are shown in yellow and light green, respectively. (c) Superimposition 3D model of the RSV prefusion protein/antibody CR9501 complexes of HDOCK and cryo-EM structure data.
