# Supplementary material for: Detailed Molecular Interactions between Respiratory Syncytial Virus Fusion Protein and the TLR4/MD-2 Complex In Silico
Source: Viruses. 2022 Oct 28;14(11):2382. doi: 10.3390/v14112382 (PMC9694959; doi:10.3390/v14112382)
Supplement: Supplementary file 1 [file viruses-14-02382-s001.zip › Supplemental Table S1.pdf]

Table S1a. Consistent molecular interactions and interacting sites of TLR4/MD-2/LPS complex in the HDOCK and X-ray crystallography data.

Interaction type	TLR4/MD-2		LPS	
Hydrogen band	TLR4	Asp294	GMH	Core oligo saccharide
		Tyr296	KDO	
	TLR4	Arg264	PO4	LipidA
	MD-2	Ser118	PO4	
Nonbonded contacts	TLR4	Asp294	GMH	Core oligo saccharide
		Tyr296	GMH	
		Tyr296	KDO	
		Arg322	PO4	
		Lys341	KDO	
		Lys341	GMH	
		Lys362	KDO	
	MD-2	Lys58	KDO	
	TLR4	Arg264	FTT	LipidA
		Arg264	PO4	
	MD-2	Val24	FTT	
		Ile46	MYR	
		Val48	FTT	
		Phe151	FTT	
		Leu54	DAO	
		Ile63	FTT	
		Ile63	MYR	
		Tyr65	FTT	
		Leu71	FTT	
		Leu74	FTT	
		Phe76	FTT	
		Phe76	MYR	
		Arg90	DAO	
		Glu92	MYR	
		Ile117	FTT	
		Ser118	PO4	
		Ser118	PO4	
		Phe119	FTT	
		Ser120	PA1	
		Ser120	FTT	
		Phe121	FTT	
		Lys122	FTT	
		Phe126	FTT	
		Ser127	FTT	
		Tyr131	FTT	
		Cys133	FTT	
		Phe151	FTT	

TLR4, Toll-like receptor 4; MD-2, Myeloid differentiation factor-2; LPS, Lipopolysaccharides; GMH, L glycero-alpha-D-manno-Heptopyranose; KDO, 3-deoxy-alpha-D-manno-oct-2-ulopyranosonic acid; PO4, Phosphoric acid; FTT, 3-Hydroxy-Tetradecanoic acid; MYR, Myristic acid; DAO, Dodecanoic acid; PA1, 2-amino-2-deoxy-alpha-D-glucophranose.

Table S1b. Inconsistent molecular interactions and interacting sites of TLR4/MD-2/LPS complex in the HDOCK and X-ray crystallography data.

Interaction type	TLR4/MD-2		LPS	
Hydrogen bonds	TLR4	Glu321	GMH	Core oligo saccharide
		Arg322	GMH	
		Lys341	GMH	
		Lys362	KDO	
	MD-2	Lys122	FTT	LipidA
Nonbonded contacts	TLR4	Arg264	PA1	Core oligo saccharide
		Tyr296	GMH	
		Tyr296	GMH	
		Thr319	KDO	
		Glu321	GMH	
		Arg322	GMH	
	MD-2	Ser120	KDO	
	MD-2	Glu92	DAO	LipidA
		Tyr102	FTT	
		Tyr102	PO4	
		Phe147	FTT	

TLR4, Toll-like receptor 4; MD-2, Myeloid differentiation factor-2; LPS, Lipopolysaccharides; GMH, L glycero-alpha-D-manno-Heptopyranose; KDO, 3-deoxy-alpha-D-manno-oct-2-ulopyranosonic acid; PO4, Phosphoric acid; FTT, 3-Hydroxy-Tetradecanoic acid; MYR, Myristic acid; DAO, Dodecanoic acid; PA1, 2-amino-2-deoxy-alpha-D-glucophranose
