# Supplementary material for: Detailed Molecular Interactions between Respiratory Syncytial Virus Fusion Protein and the TLR4/MD-2 Complex In Silico
Source: Viruses. 2022 Oct 28;14(11):2382. doi: 10.3390/v14112382 (PMC9694959; doi:10.3390/v14112382)
Supplement: Supplementary file 1 [file viruses-14-02382-s001.zip › Supplemental Table S2.pdf]

Table S2a. Consistent molecular interactions and interacting sites of RSV prefusion protein/antibody CR9501 complex in the HDOCK and Cryo-electron microscopy (cryo-EM) structure data.

Interaction type	Prefusion	Antibody CR9501	
Hydrogen bonds	Asn63	Ile100A*	Fab heavy chain
	Asn63	Ser100B*	
	Ile64	Tyr98	
	Ile64	Val99	
	Lys87	Tyr98	
	Gln94	Leu100*	
	Glu294	Ile100A*	
	Asn165	Asp28	Fab light chain
	Lys168	Tyr32	
	Asn208	Thr56	
	Glu294	Gln92	
Nonbonded contacts	Leu61	Ile100A*	Fab heavy chain
	Ser62	Ile100A*	
	Asn63	Ile100A*	
	Asn63	Val99	
	Asn63	Leu100*	
	Asn63	Ser100B*	
	Ile64	Val99	
	Ile64	Tyr98	
	Lys65	Tyr98	
	Glu66	Asp101	
	Glu66	Tyr34	
	Ile67	Tyr98	
	Asp84	Tyr98	
	Lys87	Tyr98	
	Lys87	Asp32	
	Val90	Ile100A*	
	Gln94	Leu100*	
	Lys293	Ile100A*	
	Glu294	Ile100A*	
	Glu295	Ile100A*	
	Lys65	Tyr49	Fab light chain
	Lys65	Asn53	
	Asn165	Gln92	
	Asn165	Asp28	
	Lys168	Tyr67	
	Lys168	Ser30	
	Lys168	Tyr32	

	Ser169	Tyr67	
	Leu172	Tyr67	
	Asp200	Asn53	
	Leu204	Tyr49	
	Asn208	Thr56	
	Asn208	Tyr49	
	Glu294	Tyr32	
	Glu294	Gln92	
	Glu295	Tyr32	
	Glu295	Thr31	
Salt bridges	Lys87	Asp32	Fab heavy chain

Fab, fragment antigen-binding region; RSV, respiratory syncytial virus.

* The matching amino acid sequence numbers for Ile100A, Ser100B and Leu100 reflect the Kabat numbering scheme.

Table S2b. Inconsistent molecular interactions and interacting sites of RSV prefusion protein/antibody CR9501 complex in the HDOCK and Cryo-electron microscopy (cryo-EM) structure data.

Interaction type	Prefusion	Antibody CR9501	
Hydrogen bonds	Glu66	Tyr34	Fab heavy chain
	Asp84	Tyr98	
	Lys87	Asp32	
	Glu295	Ile100A*	
	Asn208	Tyr49	Fab light chain
	Glu295	Thr31	
	Asp200	Asn53	
Nonbonded contacts	Asn63	Trp100F*	Fab heavy chain
	Ile64	Ala97	
	Lys65	Ala97	
	Lys65	Trp100F*	
	Glu66	Gly96	
	Glu66	Val2	
	Leu83	Tyr98	
	Tyr86	Val99	
	Lys87	Val99	
	Lys87	Asn33	
	Val90	Val99	
	Asn165	Tyr93	Fab light chain
	Lys196	Thr31	
	Leu204	Leu54	
	Leu204	Asn53	
	Asn208	Glu55	
	Asn208	Leu54	
	Gly162	Tyr93	
	Lys168	Thr31	

Fab, fragment antigen-binding region; RSV, respiratory syncytial virus.

* The matching amino acid sequence numbers for Ile100A and Trp100F reflect the Kabat numbering scheme.
