# Supplementary material for: Detailed Molecular Interactions between Respiratory Syncytial Virus Fusion Protein and the TLR4/MD-2 Complex In Silico
Source: Viruses. 2022 Oct 28;14(11):2382. doi: 10.3390/v14112382 (PMC9694959; doi:10.3390/v14112382)
Supplement: Supplementary file 1 [file viruses-14-02382-s001.zip › Supplemental Table S3.pdf]

Table S3. The sites with a binding probability greater than 0.5 predicted by ScanNet in prefusion and postfusion protein.

prefusion residues	postfusion residues
Gly51	Gly51
Trp52	Trp52
Tyr53	Tyr53
Thr54	Thr54
Gly139	Ser55
Phe140	Val56
Leu141	Ile57
Leu142	Thr58
Gly143	Ile59
Val144	Glu60
Gly145	Val76
Ser146	Leu78
Ala147	Gln81
Ile148	Glu82
Ala149	Lys85
Ser150	Leu96
Val152	Met97
Ala153	Lys156
Val154	Leu160
Lys156	Glu163
Val185	Val164
Ile217	Lys166
Ile221	Ile167
Gln224	Lys168
Gln225	Ala170
Asn228	Leu171
Ala241	Leu172
Val243	Ser173
Thr245	Thr174
Pro265	Asn175
Tyr286	Ala177
Pro320	Val178
Met370	Val179

Ser372	Ser180
Leu373	Leu181
Thr374	Ser182
Leu375	Gly184
Pro376	Val185
Ser398	Val187
Thr400	Leu188
Val402	Thr189
Ser404	Lys191
Ile407	Val192
Arg429	Asp194
Ile431	Leu195
Ile432	Lys196
Asn454	Tyr198
Thr455	Ile199
Leu456	Asp200
Tyr457	Gln202
Tyr458	Leu203
Asp479	Leu204
Leu481	Ile206
Val482	Val207
Ile499	Asn208
Ser502	Gln210
Leu503	Ser211
Phe505	Ile214
Ile506	Ile217
Lys508	Glu218
Ser509	Ile221
Asp510	Glu222
Leu512	Gln224
Leu513	Gln225
	Lys226
	Asn228
	Leu231
	Glu232
	Val239

Asn240
Ala241
Val243
Thr245
Pro246
Ser255
Glu256
Leu258
Ser259
Leu260
Asn262
Asp263
Pro265
Asn268
Asp269
Lys272
Ser275
Asn276
Tyr286
Ile288
Ser290
Ile291
Val296
Val300
Gln302
Ser404
Ile407
Lys421
Thr423
Ile431
Asn454
Thr455
Leu456
Tyr457
Tyr458
Lys461

Gly464
Lys465
Ser466
Leu467
Tyr468
Val469
Lys470
Gly471
Glu472
Pro473
Ile474
Ile475
Asn476
Phe477
Tyr478
Asp479
Pro480
Leu481
Val482
Phe483
Pro484
Ser485
Asp486
Glu487
Phe488
Asp489
Ser491
Ile492
Gln494
Val495
Asn496
Lys498
Ile499
Gln501
Ser502
Leu503

Phe505
Ile506
Arg507
Lys508
Ser509
Asp510
Leu512
Leu513
His514
Asn515
Val516
Asn517
